# Thermodynamic investigation of DNA-binding affinity of wild-type and mutant transcription factor RUNX1

**DOI:** 10.1371/journal.pone.0216203

**Published:** 2019-05-02

**Authors:** Fangrui Wu, Tidie Song, Yuan Yao, Yongcheng Song

**Affiliations:** Department of Pharmacology and Chemical Biology, Baylor College of Medicine, Houston, Texas, United States of America; Florida International University, UNITED STATES

## Abstract

Transcription factor RUNX1 and its binding partner CBFβ play a critical role in gene regulation for hematopoiesis. Mutations of RUNX1 cause ~10% of acute myeloid leukemia (AML) with a particularly poor prognosis. The current paradigm for the leukemogenesis is that insufficient activity of wild-type (WT) RUNX1 impairs hematopoietic differentiation. The majority of mutant RUNX1 proteins lose the DNA-binding affinity and inhibit WT RUNX1 by depletion of CBFβ. Here, isothermal titration calorimetry (ITC) was used to quantitatively study the interactions of WT and three clinical mutant RUNX1, CBFβ and DNA. Our data show that the binding of RUNX1 to DNA is enthalpy-driven, and the affinity decreases in the order of WT > S114L > R139Q >> K83E, which support previous observations and conclusion. To find potentially beneficial RUNX1 mutations that could increase the overall RUNX1 activity, K83R and H179K mutations of RUNX1 were designed, using structure-based computational modeling, and found to possess significantly higher DNA-binding affinities than does WT RUNX1. K83R and H179K mutant RUNX1 could therefore be protein-based RUNX1 activators.

## Introduction

**Fig 1:**
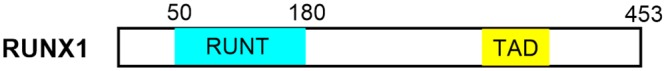
RUNX1 contains the RUNT domain and transactivation domain (TAD).

Hematopoiesis, generating ~10^11-12^ differentiated blood cells daily in humans, requires exquisite gene expression regulation. Dysfunction of these controls could lead to initiation of a blood disease, or even leukemia. Transcription factor RUNX1 (also known as AML1) [1] is a master regulator for hematopoiesis [2,3]. It belongs to the core binding factor (CBF) transcription factor family, which consists of CBFα (including RUNX1, 2 and 3) and CBFβ proteins. RUNX1 contains an N-terminal RUNT domain (50–180), which is highly conserved from yeast to mammals, and C-terminal transactivation domains (Fig 1) [4,5]. The biological function of RUNX1 is that RUNT recognizes and binds to the promoter of its target genes with a consensus sequence of PyGPyGGTPy [6–9], with the transactivation domains recruiting other transcriptional proteins (e.g., p300) to activate or repress transcription of the gene [2,10–13]. While CBFβ does not interact with DNA, it forms a heterodimeric complex with RUNT, which can significantly increase the DNA-binding affinity [7] and is essential for hematopoiesis [14,15]. This suggests high affinity binding of RUNX1 to DNA is crucial: reduction of its DNA-binding affinity (e.g., loss of CBFβ) may block the RUNX1-mediated gene transcription and cause abnormal hematopoiesis.

Somatic and germline mutations of RUNX1 cause ~10% of acute myeloid leukemia (AML) as well as preleukemic diseases with predisposition to AML, such as myelodysplastic syndrome (MDS) (~10%) [16–18]. There are rare cases of inherited, heterozygous germline RUNX1 mutations, which cause familial platelet disorder (FPD) showing low counts or functional defects of platelets [19–21]. Depending on the nature of the mutations, 20%-57% of these FPD patients develop AML at the age of 8–62. RUNX1 mutated AML patients have a particularly poor prognosis [22,23] with significantly lower rates of complete remission (47%) and 5-year survival (2%) [22], as compared with those without RUNX1 mutation (77% and 30%, respectively).

The current paradigm for RUNX1 mutation-mediated leukemia initiation is due to insufficient activity of wild-type (WT) RUNX1 for hematopoiesis [16–18], which causes accumulation of immature blood cells as well as insufficient numbers of functional blood cells. Over the time, acquiring a second (or more) mutation could lead to an onset of leukemia. It is noteworthy that even RUNX1 haploinsufficiency could lead to AML. A pedigree of FPD patients exhibit RUNX1^+/-^ genotype with one RUNX1 allele deleted [19,20]. The haploinsufficiency caused 7 out of 24 patients to develop leukemia. >80% of RUNX1 mutations occur in the RUNT domain with R80, K83, S114, R139, D171, R174 and R177 being hot spots. Except for S114 which is located in the interface with CBFβ, these residues have interactions with DNA [7]. This suggests that these mutations affect RUNX1’s binding ability to DNA. Several leukemia-causing mutations, such as K83E, R139Q and R174Q, were found to have a reduced DNA binding affinity, but they retain the ability to bind CBFβ [21,23–26]. Thus, these RUNX1 mutants could inhibit the activity of WT RUNX1 by reducing the amount of CBFβ. The overall levels of RUNX1 activity seem to correlate with the predisposition to leukemia [26,27].

However, previous studies of the interactions of these clinical RUNX1 mutants with DNA and CBFβ were based on less quantitative electrophoretic mobility shift assay (EMSA). More accurate characterization of the binding affinity of mutant RUNX1 to DNA is needed. Here, we report results of thermodynamic studies of interactions between RUNX1, CBFβ and DNA, using isothermal titration calorimetry (ITC). WT and 3 selected leukemia-causing mutant (i.e., K83E, S114L and R139Q) RUNX1 were included. In addition, we hypothesize that a “good” mutant RUNX1 with a higher binding affinity to DNA could counteract a leukemia-causing RUNX1 mutant and have an enhanced RUNX1 activity for hematopoiesis. This could be a therapeutic approach to AML with RUNX1 mutation. We report 3 novel RUNX1 mutant proteins that were designed using computational modeling and possess a significantly higher binding affinity to DNA than does the WT RUNX1.

## Material and methods

### Constructs for recombinant proteins

Human RUNX1 gene containing amino acid sequence 1–181 and CBFβ (1–141) were cloned and inserted into pET-28α expression vector. The correctness of the inserted DNA was verified by sequencing.

### Site-directed mutagenesis

Site-directed mutagenesis was performed to produce mutant forms of RUNX1, using QuikChange Mutagenesis kit (Agilent) following the manufacturer’s instructions. Within RUNX1, the mutations K83E, S114L, R139Q, K83R, H179K were made individually by using PCR. Two consecutive site-directed mutagenesis experiments were used to yield the K83R/H179K double mutation. The forward primer (5'- ATGGGCAGGGTCTCGTTGCAGCGCCAG -3’) and backward primer (5'- CTGGCGCTGCAACGAGACCCTGCCCAT -3') for K83E, the forward primer (5'- CTCAGCTCAGCCAAGTAGTTTTCATCATTGCCAGCC -3’) and backward primer (5'- GGCTGGCAATGATGAAAACTACTTGGCTGAGCTGAG -3') for S114L, the forward primer (5'- TTTTCCCTCTTCCACTTTGACCGACAAACCTGAGG -3’) and backward primer (5'- CCTCAGGTTTGTCGGTCAAAGTGGAAGAGGGAAAA -3') for R139Q, the forward primer (5'-GTTCACTGCCGCTTTCTTCGAGGTTCTCGGGGCCC-3’) and backward primer (5'-GGGCCCCGAGAACCTCGAAGAAAGCGGCAGTGAAC-3') for H179K and the forward primer (5’-GATGGGCAGGGTCCTGTTGCAGCGCCA-3’) and backward primer (5’-TGG CGCTGCAACAGGACCCTGCCCATC-3’) for K83R were designed with the QuikChange Primer Design Program at Agilent Genomics (http://www.genomics.agilent.com). Sequences of all inserted genes were verified by sequencing.

### Protein expression and purification

BL21-CodonPlus strain (Agilent) was transformed with pET28-RUNX1 plasmid using heat shock and cultured at 37 °C in LB medium containing ampicillin (50 μg/mL) and Kanamycin (50 μg/mL). Upon reaching an optical density of ~1.3 at 600 nm, RUNX1(1–181) expression was induced by adding 0.2 mM isopropylthiogalactoside (IPTG) at 18 °C for 20 hours. Cells were harvested, lysed, centrifuged at 20,000 rpm for 20 min and the supernatant was collected and subjected to an affinity column chromatography using a Ni-NTA column (GE Healthcare) followed by a Superdex 75 gel filtration column. All recombinant proteins were found with purity of >90% (SDS-PAGE).

### Isothermal titration calorimetry

Isothermal titration calorimetry (ITC) was used to measure the interaction between RUNX1 and 21 base-pair DNA fragment (5’-CAAACTCTGTGGTTGCCTTGC-3’) derived from human CSF-1R gene promoter. The ITC experiments were performed using a Nano ITC LV (190 μL) instrument from TA Instruments (New Castle, DE) at 277 K. A 190 μL solution containing 20 μM Runx1 protein in 10 mM HEPES-NaOH buffer (pH 7.5), containing 0.18 M NaCl and 0.005% Tween 20 was added into the sample cell. DNA fragment (100 μM) in the same buffer solution (50 μL) was transferred into the syringe of the instrument. Upon reaching temperature equilibrium, 25 injections (2 μL each) of the inhibitor were used for the titration. ITC data were then imported into NanoAnalyze software (TA Instruments, New Castle, DE). The titration baseline was corrected, and the ΔH and *K*_d_ values for the binding of the inhibitor were obtained by fitting into the independent model in the software. Three independent experiments were done to ensure that the reported data are reliable.

### Molecular modeling

Molecular modeling studies were performed using software package Schrödinger suite (version 2018, Schrödinger LLC, NY), which includes all of the programs described below. The structure of DNA-RUNX1-CBFβ complex were prepared using the module “protein preparation wizard” in Maestro with default protein parameters, using the X-ray crystal structure 1IO4 (PDB code) [7] available in Protein Data Bank. Hydrogen atoms were added and irrelevant ligands and all water molecules were removed. Next, hydrogen bonds were optimized, the partial charges for all atoms were assigned, and the protein-DNA complex was energy-minimized using OPLS-2005 force field. Hydrophobic, electrostatic and H-bond interactions among DNA, RUNX1 and CBFβ can be visualized in Maestro. A RUNX1 point mutation (such as K83R) was generated manually by deleting the original sidechain followed by adding a new sidechain in Maestro. The resulting mutant protein structure was globally energy-minimized using OPLS-2005 force field. All figures for molecular modeling studies were generated using Maestro.

## Results

### Leukemia-causing RUNX1 mutants have reduced DNA-binding affinity

Isothermal titration calorimetry (ITC) was used to quantitatively measure the DNA-binding affinity of WT and 3 selected clinical mutant RUNX1 proteins. In addition, thermodynamic data including changes in enthalpy and entropy (ΔH and ΔS) for the protein-DNA interactions were determined. Because the full-length RUNX1 was not expressed well in *E*. *coli* due to its low stability and high propensity to aggregation, the N-terminal 1–181 protein that includes the DNA-binding RUNT domain was used for the study. Three hotspot mutations K83E, S114L and R139Q in AML were chosen and generated with site-specific mutagenesis from the WT RUNX1. All of these recombinant proteins with an N-terminal His6-tag were expressed in *E*. *coli* (BL21-CodonPlus strain) and purified with affinity and size-exclusion chromatography in a high yield (~20 mg/L culture) and >90% purity (SDS-PAGE). Recombinant CBFβ (1–141) protein was obtained similarly.

**Fig 2:**
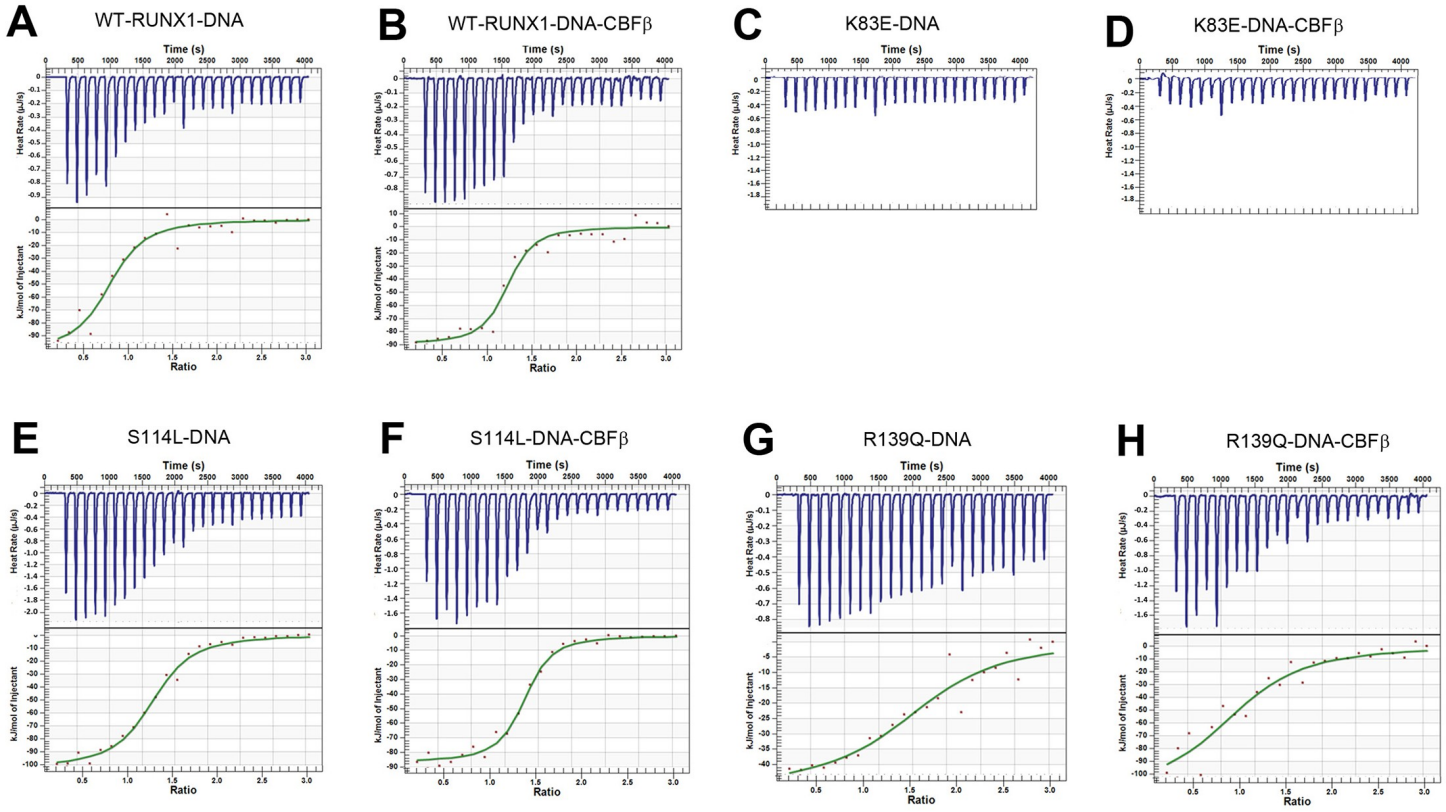
Representative ITC results and fitting curves for titration of DNA into WT and clinical mutant RUNX1, using the independent model.

A 21 base-pair DNA (5’-CAAACTCTGTGGTTGCCTTGC-3’) from human CSF-1R gene promoter was used for the studies of RUNX1-DNA interactions [7]. ITC experiments were performed by titrating the DNA solution (100 μM, 25 injections of 2 μL each) into the RUNX1 protein solution (20 μM, 190 μL) at 25 °C in 10 mM HEPES buffer (pH 7.5) containing 180 mM NaCl and 0.005% Tween 20. For experiments including CBFβ, a premixed RUNX1-CBFβ (1:2) solution was used. The ITC results are shown and summarized in Fig 2 and Table 1.

**Table 1:** Thermodynamics data for RUNX1-DNA interactions.

RUNX1	CBFβ	Δ*H* (kJ/mol)	*K*_d_ (μM)	Δ*G* (kJ/mol)	Δ*S* (J K^-1^ mol^-1^)
WT	-	-99.3 ± 4.2	0.49 ± 0.01	-42.2	-206
	+	-81.8 ± 9.0	0.23 ± 0.05	-34.7	-170
K83E	-	ND^a^	No binding	ND^a^	ND^a^
	+	ND^a^	No binding	ND^a^	ND^a^
S114L	-	-100.0 ± 15.4	0.70 ± 0.08	-33.0	-242
	+	-61.8 ± 7.5	0.39 ± 0.08	-33.5	-102
R139Q	-	-54.4 ± 39.3	1.48 ± 0.51	-29.7	-89
	+	-100.0 ± 16.7	3.35 ± 0.8	-30.5	-251
K83R	-	-69.4 ± 5.6	0.48 ± 0.15	-32.8	-132
	+	-64.1 ± 2.9	0.16 ± 0.03	-35.6	-103
H179K	-	-63.1 ± 7.4	0.35 ± 0.12	-33.5	-107
	+	-100.0 ± 6.5	0.10 ± 0.04	-31.0	-249
K83R/H179K	-	-100.0 ± 11.0	0.29 ± 0.05	-34.4	-237
	+	-84.7 ± 9.1	0.073 ± 0.005	-43.7	-148

^a^ND: not determined.

In the absence of CBFβ, the binding of WT RUNX1 to DNA was found to be strongly exothermic, with a change in enthalpy (Δ*H*) of -99.3 ± 4.2 kJ/mol (Fig 2A). Fitting the titration curve using the independent model gave the DNA-binding affinity of WT RUNX1 with a *K*_d_ (dissociation constant) value of 0.49 ± 0.01 μM, which equals a change in free energy (Δ*G*) of -42.2 kJ/mol. With a calculated change in entropy (Δ*S*) of -206 J K^-1^ mol^-1^, the experiment shows the binding of WT RUNX1 to DNA is enthalpy-driven. CBFβ was found to significantly enhance the binding affinity of WT RUNX1 to DNA by ~2-fold, with a *K*_d_ value of 0.23 ± 0.05 μM (Δ*G* = -34.7 kJ/mol) (Fig 2B and Table 1). The binding is also highly exothermic (Δ*H* = -81.8 ± 9.0 kJ/mol) and enthalpy-driven. In contrast, titration of DNA into the K83E mutant RUNX1 (abbreviated to be K83E) with or without CBFβ did not generate a significant amount of enthalpy change (Fig 2C and 2D), suggesting K83E does not bind to DNA. Although this observation does not exclude the possibility that K83E binds to DNA without an enthalpy change, its lack of DNA-binding ability is consistent with previous EMSA results [21]. DNA-binding of S114L mutant RUNX1 exhibited a large Δ*H* of -100.0 ± 15.4 kJ/mol and a reduced DNA-binding affinity with a *K*_d_ value of 0.70 ± 0.08 μM, as compared with WT RUNX1. Although CBFβ was reported to bind significantly weaker to S114L (which is in the interface between RUNX1 and CBFβ) [7], CBFβ was again found to enhance the DNA-binding affinity of S114L by ~2x, with a *K*_d_ value of 0.39 ± 0.08 μM. Nonetheless, ITC investigation showed that as compared to WT RUNX1, S114L possesses ~2-fold reduced affinity to DNA. Titration of DNA to the R139Q mutant RUNX1 with and without CBFβ were exothermic and gave *K*_d_ values of 1.48 ± 0.51 and 3.35 ± 0.8 μM, respectively, showing that R139Q mutation exerts a more pronounced effect to weaken the DNA-binding ability, and complexation with CBFβ does not improve the binding.

Overall, our ITC experiments demonstrate that the binding of RUNX1 to DNA is enthalpy-driven, and the binding affinity decreases in the order of WT > S114L > R139Q >> K83E. In a quantitative manner, these data are in line with previous EMSA results, supporting the correlation between reduced DNA-binding affinity, decreased RUNX1 activity in gene regulation, and increased predisposition to leukemia in the clinic.

### Computational design of RUNX1 mutants with enhanced DNA-binding affinity

We hypothesize that a RUNX1 mutation that binds to DNA stronger could counteract those leukemia-causing RUNX1 mutations. However, no mutant RUNX1 with an enhanced DNA-binding ability has been reported. Molecular modeling software Schrödinger suite was used to design such RUNX1 mutations. Using the X-ray structure of a DNA-RUNX1-CBFβ complex (PDB code 1IO4) [7] in the Protein Data Bank as a template, the structure of RUNX1-DNA-CBFβ was prepared and visualized. Structural analysis revealed that the high-affinity binding of RUNX1 to DNA mainly depends on numerous favorable hydrogen bond and electrostatic interactions. This observation is in line with our ITC studies showing the binding of RUNX1 to DNA is enthalpy-driven (Table 1). Increasing hydrogen bond, electrostatic and/or other polar interactions between RUNX1 and DNA could lead to a higher binding affinity.

**Fig 3:**
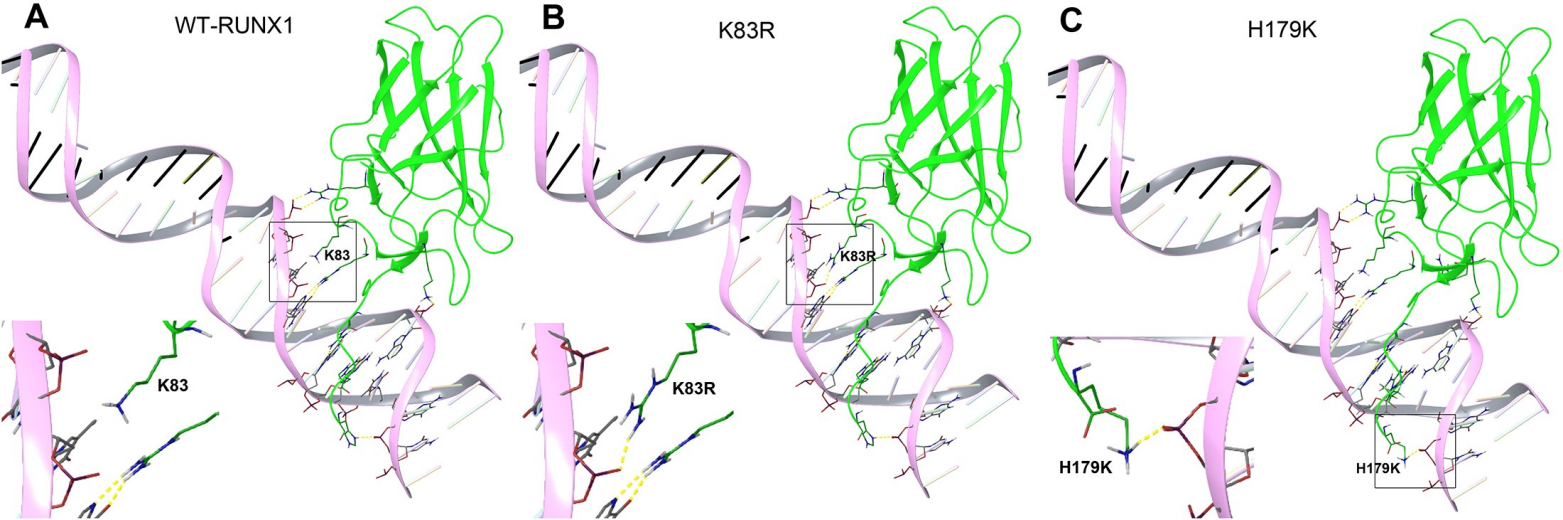
(A) X-ray structure of WT RUNX1 in complex with DNA (PDB: 1IO4), with DNA shown as a pink model and RUNX1 in green. For clarity, only interacting residues are shown. Hydrogen bonds are shown as yellow dashed lines; (B) Modeled structures of the K83R RUNX1-DNA complex, showing favorable hydrogen-bond/electrostatic interactions between mutated K83R and DNA phosphate; (C) Modeled structures of the H179K RUNX1-DNA complex, showing favorable hydrogen-bond/electrostatic interactions between mutated H179K and DNA phosphate. The bottom-left insets show the enlarged areas in the black boxes.

There are several opportunities for a RUNX1 point mutation to have increased polar interactions with DNA. One possibility is between the sidechain of K83 and DNA. In the crystal structure, there is no direct interactions between K83 and a DNA’s phosphate (Fig 3A). Rather, a water molecule is found as a bridge to form two hydrogen bonds between K83 and DNA [7]. Thus, the point mutation K83R was designed with a rationale that Arg has a longer, positively charged side chain, which could provide direct and possibly stronger interactions with the DNA’s phosphate group. Next, K83R mutant RUNX1 was generated in the computer and globally energy-minimized using OPLS-2005 force field. The optimized conformation of K83R side chain was predicted to have favorable hydrogen bond and electrostatic interactions with the DNA backbone phosphate group (Fig 3B). Similarly, His-179 sidechain was found to have a hydrogen bond with a nearby DNA phosphate group (Fig 3A). We reasoned that a positively charged lysine sidechain could have improved polar interactions. Using a similar method, H179K mutant RUNX1 was generated and modeled. Modeling results show hydrogen bond and electrostatic interactions between the positively charged lysine and DNA are expected (Fig 3c), which have the potential to increase the binding affinity.

### K83R and H179K RUNX1 mutants have a higher affinity to DNA

**Fig 4:**
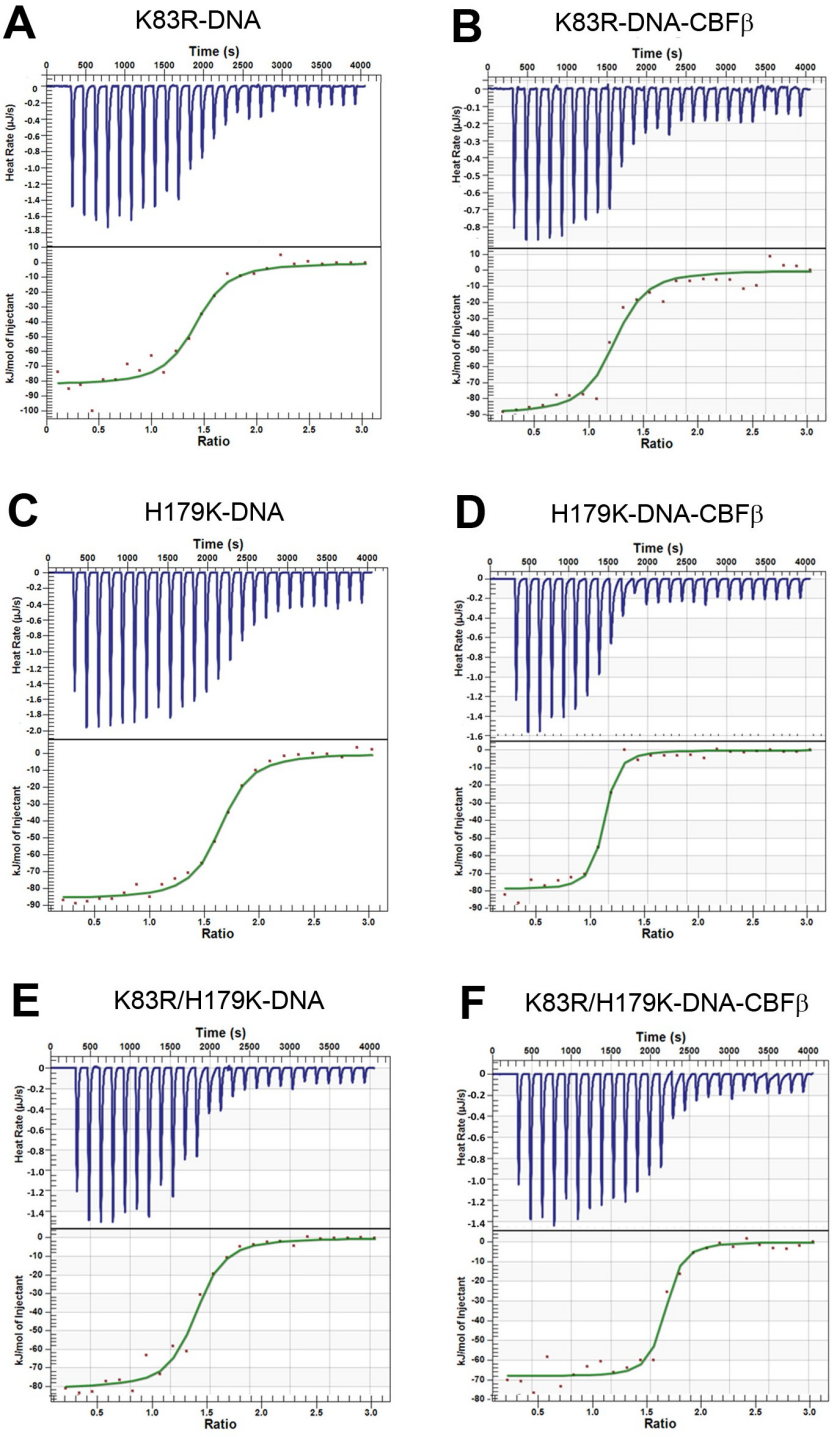
Representative ITC results and fitting curves, using the independent model, for titration of DNA into mutant RUNX1 proteins that are designed to have a potentially improved binding affinity.

Through site-directed mutagenesis followed by protein expression and purification, recombinant RUNX1 proteins with K83R, H179K and double K83R/H179K mutations were obtained. Their binding affinities to DNA in the presence or absence of CBFβ were determined with ITC and the results are shown in Fig 4 and Table 1. In the absence of CBFβ, titration of DNA into K83R mutant RUNX1 was found to be an exothermic reaction, giving Δ*H* of -69.4 ± 5.6 kJ/mol and *K*_d_ of 0.48 ± 0.15 μM (Fig 4A). CBFβ was found to also significantly enhance the binding affinity of K83R by 3-fold with a *K*_d_ value of 0.16 ± 0.03 μM (Fig 4B). K83R mutation showed a comparable or slightly higher binding affinity, as compared with WT RUNX1 (*K*_d_ = 0.49 or 0.23 μM with or without CBFβ, respectively). The H179K mutation was found to exert a more pronounced improvement. H179K alone binds to DNA with a *K*_d_ value of 0.35 ± 0.12 μM (Fig 4C), more tightly than WT RUNX1. When complexed with CBFβ, H179K showed a strong binding ability with a *K*_d_ of 100 ± 40 nM (Fig 4D), having ~2× more affinity to DNA than does the WT RUNX1/CBFβ. In addition, the K83R/H179K double mutant RUNX1 exhibited an improved binding affinity as compared to that of the H179K mutant, with the *K*_d_ values of 0.29 ± 0.05 and 0.073 ± 0.005 μM with and without CBFβ, respectively (Fig 4E and 4F).

## Discussion

Transcription factor RUNX1, together with its partner CBFβ, plays a critical role in gene expression regulation in hematopoiesis. RUNX1 gene mutations are frequently found in acute myeloid leukemia (AML) with a particularly poor prognosis. There have been no effective treatments for this subtype of AML. In addition, RUNX1 mutations are also commonly found in a number of pre-leukemic diseases, such as myelodysplastic syndrome (MDS) and family platelet disorders (FPD), with predisposition to AML, showing RUNX1 mutation is an early event and the driving force to the cancer. The current paradigm of the mechanism underlying the malignancy is due to insufficient overall biological activity of WT RUNX1, which stems from haploinsufficiency or a mutant RUNX1 that either reduces the amount of CBFβ (for N-terminal mutations) or lacks the transactivation activity (for C-terminal mutations). Insufficient activity of RUNX1 causes hampered hemopoietic differentiation, accumulation of undifferentiated blood cells, and eventually, upon acquisition of other mutations, onset of the leukemia.

High-affinity binding of RUNX1 to its target DNA is critical to the activity of RUNX1. Complexation with CBFβ, which can significantly enhance the binding affinity of RUNX1, is required for hematopoiesis. Therefore, isothermal titration calorimetry (ITC) was used in this study to quantitatively measure the interactions of WT and three clinical mutant RUNX1, CBFβ and DNA. Our data show that the binding of RUNX1 to DNA is enthalpy-driven, and the binding affinity decreases in the order of WT > S114L > R139Q with *K*_d_ values of 0.49, 0.70, 1.48 μM, respectively. K83E mutant does not bind to DNA. In addition, CBFβ was found to cause ~2-fold increased binding affinity of WT and S114L RUNX1 with *K*_d_ values of 0.23 and 0.39 μM, while CBFβ reduces the bind affinity of R139Q. In the presence of CBFβ, K83E still had no interactions with DNA. These results complement and are generally consistent with those in previous studies using electrophoretic mobility shift assay (EMSA), showing that as compared with WT RUNX1, leukemia-causing RUNX1 mutants have a significantly reduced binding affinity to DNA. As a consequence, the overall activity of RUNX1 in the cancer cells is decreased.

An interesting question we had next is whether there is a mutant RUNX1 bearing a higher DNA-binding ability than the WT protein. Such a “good” mutant could provide an elevated RUNX1 activity and counteract a leukemia-causing RUNX1 mutation. Using structure-based computational modeling, two RUNX1 mutations K83R and H179K were designed with a rationale that more hydrogen-bond and electrostatic interactions with DNA could increase the binding affinity. ITC investigation showed that in the absence of CBFβ, K83R mutant protein possesses a comparable binding affinity, while H179K can significantly enhance DNA-binding affinity. When complexed with CBFβ, more pronounced effects were observed with *K*_d_ values for K83R and H179K being 0.16 and 0.10 μM. Compared to that of H179K, RUNX1 with double mutations of K83R and H179K exhibited an improved DNA-binding affinity. Lysine-83 is shown to play an important role in binding to DNA. The leukemia-causing K83E mutation was found to completely abrogate the DNA-binding ability of the protein, because of the electrostatic repulsion between the negatively charged sidechain of glutamate and the DNA phosphate. Our designed K83R mutation, which is positively charged and has a longer sidechain, could form direct hydrogen bond and electrostatic interactions with the DNA-phosphate (without needing a bridging water molecule) (Fig 3B). Histidine-179 lies on the outer edge of the RUNT domain. H179K mutation, which replaces the mostly neutral histidine sidechain with a positively charged lysine sidechain, also greatly increases the affinity to DNA, likely through more favorable hydrogen-bond and electrostatic interactions with the DNA-phosphate.

Possible benefits for introducing a “good” RUNX1 mutation with a higher DNA-binding ability, such as H179K, are two-folded. First, the “good” mutant (with active C-terminal transaction domains) increases the net amount of the active form of RUNX1, thereby enhancing the overall RUNX1 activity. Second, there is a possibility that the activity of such a “good” RUNX1 is independent of CBFβ. For example, the DNA-binding affinity of K83R/H179K alone (*K*_d_ = 0.29 μM) is comparable to that of the WT RUNX1-CBFβ complex (*K*_d_ = 0.23 μM). Thus, the beneficial RUNX1 mutant could counteract a leukemia-causing RUNX1 that reduces the amount of CBFβ. Therefore, H179K/K83R mutant RUNX1 could serve as a protein-based RUNX1 activator, having the potential to boost RUNX1 transactivation ability and correct the leukemic phenotype.

In summary, isothermal titration calorimetry (ITC) investigation of DNA-binding ability of the WT and clinical mutations of RUNX1 quantitatively confirms results from earlier research [21,23–26], showing that DNA-binding of the leukemia-relevant mutant RUNX1 is significantly decreased or lost. Our hypothesis is a mutant RUNX1 with a significantly higher DNA-binding affinity could overcome leukemia-RUNX1 mutations and restore RUNX1-mediated hematopoiesis. Towards achieving this goal, K83R and H179K RUNX1 mutations have been identified to have a significantly enhanced affinity to DNA. Our future work is to develop a cell-based assay to evaluate cellular activity of RUNX1 and test whether transfecting K83R or H179K RUNX1 into the cells would lead to significantly increased RUNX1-mediated gene expression. The ultimate goal is to develop a RUNX1 activation therapy for treatment of RUNX1 mutated leukemia.
